# A One-Pot Fabrication of Chitosan Gel-Encapsulated Gold Nanoparticles Using Inkjet Mixing Technology

**DOI:** 10.1021/acsomega.5c03957

**Published:** 2025-09-04

**Authors:** Yosuke Muranaka, Yukako Nishimuro, Taisuke Maki

**Affiliations:** Department of Chemical Engineering, 12918 Kyoto University, Kyoto 615-8510, Japan

## Abstract

The one-pot synthesis of chitosan gel-encapsulated gold nanoparticles (AuNPs) was investigated by using an inkjet mixing system. Through the collision of microdroplets, AuNPs with an average diameter of 5.1 nm and a coefficient of variation of 0.19 were successfully synthesized via a reduction method. In the synthesis of chitosan capsules, it was found that the type of gelling agent influences the diameter and particle size distribution of the resulting capsules due to their reaction rates. Utilizing the advantages of the inkjet mixing system, the one-pot synthesis of chitosan gel-encapsulated AuNPs was successfully achieved. It was found that the time between the nucleation of AuNPs and gelation was critical for encapsulation. It was thus demonstrated that the potential of the inkjet mixing system can be expanded to the synthesis of various types of nanoscale products by designing the process and that the system can be a powerful tool to reveal the mechanism of nanoparticle synthesis by precisely tuning the conditions.

## Graphical abstract

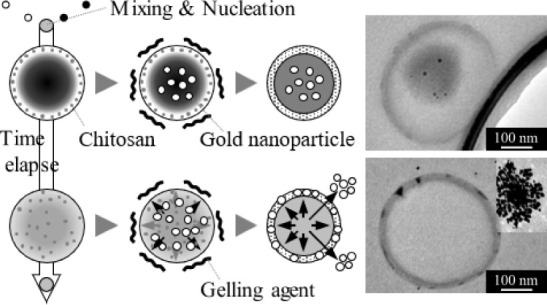

## 1 Introduction

Gold nanoparticles (AuNPs) have excellent inherent optoelectronic properties due to their interaction with visible light and are used in various fields, including organic solar cells,^1^ conductive materials,^2^ and catalysts.^3,4^ In the biological field, they serve multiple purposes, such as in photothermal therapy and biosensing.^5^ The interaction between AuNPs and light is strongly affected by their particle size and morphology.^6,7^ For AuNPs measuring several tens of nanometers, the surface plasmon resonance phenomenon leads to the absorption of light in the blue to green region of the spectrum and the reflection of red light, resulting in a red appearance. As the particle size increases, the resonance wavelength shifts toward longer wavelengths, causing red light to be absorbed and blue light to be reflected, which gives a purple color. As the particle size increases further, the resonance wavelength shifts into the infrared region, causing most visible light to be reflected and rendering the AuNPs transparent. In this way, AuNPs with optical properties suitable for various applications can be produced by changing their particle sizes and shapes. During the synthesis of AuNPs, gold precursor ions are reduced, and nucleation begins when the solution reaches a supersaturated state.^8^ To prevent particle aggregation, it is effective to add a protective agent that adequately protects each synthesized fine particle.^9^ Therefore, high-speed mixing is important for the synthesis of small, monodisperse AuNPs.

Inkjet technology involves the ejection of microdroplets from an inkjet head and enables the stable and precise discharge of extremely small droplets, even below picoliters.^10^ This technology extends beyond conventional printing on paper and is applied across many industrial fields, including electronics and biotechnology. For example, it is widely used in fine particle synthesis,^11,12^ 3D modeling,^13^ bioprinting,^14^ etc. From an inkjet nozzle, microdroplets can be continuously ejected with high reproducibility in volume, position, and speed at a regular frequency. A reaction can be initiated by colliding reaction materials in droplets, and the merged tiny droplet can serve as an individually separated tiny reactor with high reproducibility, which is a suitable characteristic for nanoparticle synthesis. In addition, rapid mixing can be achieved by colliding two droplets ejected from two separate nozzles, as the maximum diffusion distance is confined within the tiny droplet diameter.^15^

To utilize the synthesized AuNPs, encapsulating nanoparticles in polymers has been studied to improve stability and enable controlled release.^16^ In particular, the main advantage of encapsulating AuNPs in polymers is that it preserves the optical properties of AuNPs by suppressing aggregation and preventing undesirable shifts in the absorption wavelength. Chitosan capsules, which use the biocompatible polymer chitosan as a membrane material, have been widely studied in biology, including drug delivery.^17^ The structure of chitosan capsules is a 3D cross-linked network in which polymer chains are linked by cross-linkers. Various techniques are used to gel chitosan, including the reverse micelle method,^18^ emulsification solvent diffusion,^19^ and coacervation,^20^ depending on the desired properties. Ionic cross-linking, one of the cross-linking methods, is a mechanism in which chitosan capsules are formed through ionic gelation, which occurs due to electrostatic interactions between the positively charged amino groups of chitosan and negatively charged cross-linkers, such as tripolyphosphate (Figure 1). This process offers the advantages of being simple and mild, and can be performed without the use of harmful organic solvents Artiga et al. proposed a method for the production of encapsulated AuNPs in highly biocompatible chitosan hydrogels using inkjet technology.^21^ They successfully synthesized spherical, homogeneous chitosan gel-encapsulated AuNPs by ejecting a chitosan solution containing AuNPs from an inkjet nozzle and dropping it into a 60% ethanol solution containing phosphotungstic acid (PTA) as a gelling agent. Dohnal et al. demonstrated that inkjet technology can be used to prepare calcium alginate hydrogel microcapsules containing TiO_2_ photocatalysts.^22^ Encapsulation was performed by dropping a solution containing TiO_2_ solid particles into an inkjet head. Their research is outstanding and will make a significant contribution to the production of capsules containing nanoparticles. However, all of the methods they have examined involve ejecting droplets of a gel precursor that already contains nanoparticle and then gelling them in a collection tray. This method poses risks, for example, nanoparticle aggregation and, most importantly, nozzle clogging. The aim of this study was to overcome these risks by examining a one-through synthesis and encapsulation process. This is expected to lead to the production of a wider variety of capsules containing nanoparticles in different sizes and materials. In this study, chitosan gel-encapsulated AuNPs were selected as the target product.

**Figure 1:**
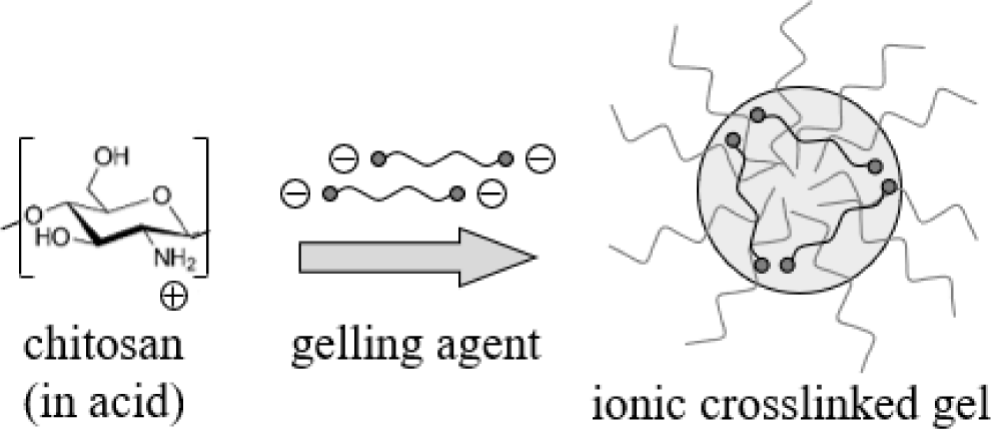
Gelation mechanism of chitosan by ionic cross-linking.

## 2 Materials and Methods

### 2.1 Materials

A 1 mM tetrachloroauric­(III) acid solution and a 20 mM chitosan solution in 1 wt % acetic acid were used as the gold precursor solution. Chitosan was selected for its dual properties: as a gel material and as a protective agent against AuNP aggregation. A 10 mM citric acid solution in deionized water was used as the reducing agent. As gelling agents, 2.3 mM sodium tripolyphosphate (TPP) in 1 wt % acetic acid and 10 mg/mL PTA hydrate in 60 vol % ethanol were examined. All chemicals were purchased from Fujifilm Wako Chemicals (Japan).

### 2.2 Apparatus, Procedure, and Analysis

Figure 2 shows the conceptual diagram of the apparatus used in this study. The inkjet mixing apparatus (IJK-200 W) was fabricated by Microjet Corp. (Nagano, Japan). Two opposing nozzles independently discharged small droplets through the shape change of a piezoelectric element in response to voltage. The discharged droplets collided and merged in the air. AuNP nucleation and particle growth occurred in the merged droplets via reduction, and encapsulation proceeded in the reservoir collection vial. The nozzles discharged droplets by using a piezoelectric system, where the piezoelectric elements changed their volume in response to voltage. The diameter of the discharged droplets was set at 60 μm. The distance between the collision point and the collection vial was initially set to 8.5 cm and was adjusted between 3–15 cm by elevating the collection vial using a laboratory jack. Chitosan gel capsule synthesis was also attempted using single-nozzle droplet ejection to examine the type of gelling agent. The droplet ejection frequency was set to 500 Hz for all experiments. The synthesized AuNPs and chitosan gel capsules were photographed by using a transmission electron microscope (TEM) (JEM-1010, JEOL, Japan). Particle sizes were measured from the TEM images using ImageJ.^23^ The optical properties of the synthesized chitosan gel-encapsulated AuNPs were measured by UV–vis–NIR spectrometry using a UV–vis–NIR spectrophotometer (V-730, JASCO, Japan). To ensure that the properties of the synthesized chitosan gel-encapsulated AuNPs did not change after collection, the analysis was repeated 1 week after the experiments using the remaining product solutions. The temperature was set at 25 °C during the experimental and storage processes.

**Figure 2:**
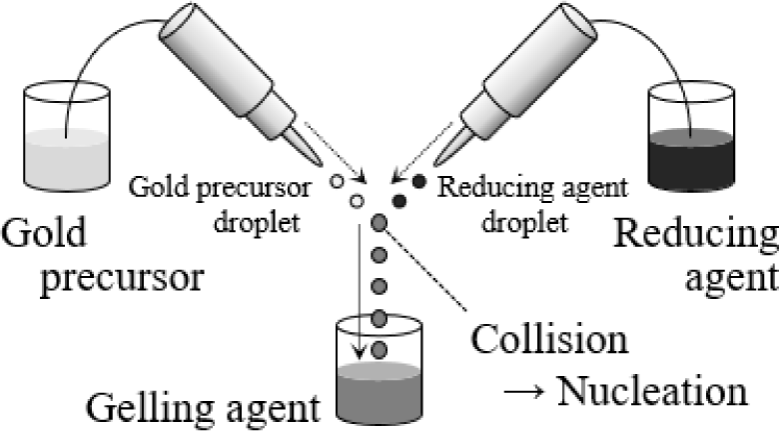
Conceptual diagram of the experimental setup.

## 3 Results and Discussion

### 3.1 Synthesis of AuNPs and Chitosan Gel Capsules

First, the synthesis of AuNPs alone was examined via reduction by a colliding gold precursor solution and a citric acid solution. An example of a TEM image is shown in Figure 3. As shown in Figure 3a, most of the particles appeared monodisperse, with sizes ranging from a few nanometers. However, as shown in Figure 3b, in some areas with a high particle density, several particles had aggregated. Particle size analysis was performed by counting the primary particles within the aggregates, and the results are shown in Figure 3c. The average diameter, *d*_av_, was approximately 5 nm, with a coefficient of variation (CV) of 0.19, indicating that monodisperse AuNPs were successfully synthesized using the inkjet mixing system. Huang et al. have previously investigated the effect of chitosan concentration and molecular weight as a protective agent on AuNP synthesis.^24^ It should be noted that, in their study, chitosan was simply used as a protective agent, not as a capsule material. Their report found that the properties of chitosan did not significantly affect particle size but did affect stability. At low chitosan concentrations, particles aggregated, whereas at high concentrations, particles remained stable for at least two months. Because chitosan is also expected to serve as a capsule material in this study, a concentration that is too low is not desirable. In other words, a certain concentration is necessary to stabilize particles and form capsules, but this does not have a significant effect on the AuNP particle size.

**Figure 3:**
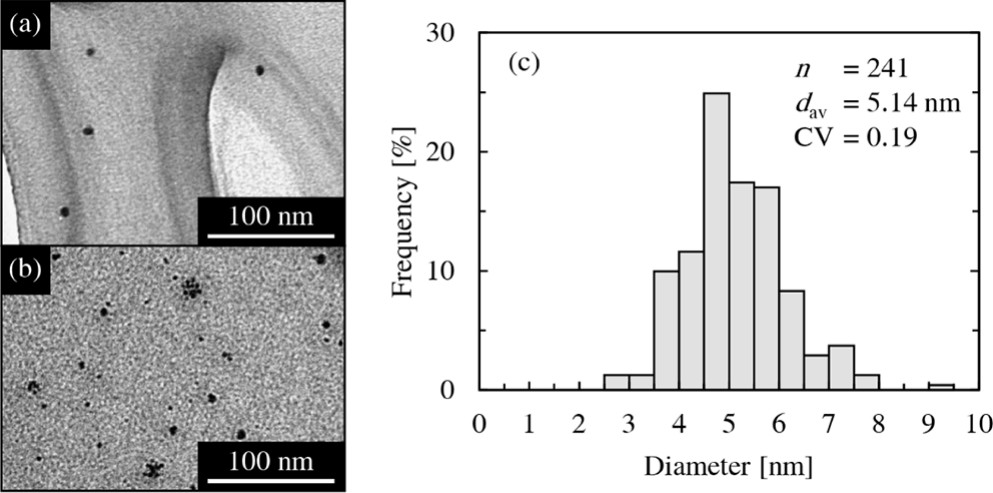
(a,b) TEM images; (c) particle size distribution of AuNPs synthesized using the inkjet mixing system.

Next, the synthesis of chitosan gel capsules was examined. The particle size distributions and TEM images of chitosan capsules prepared using two types of gelling agents are shown in Figure 4. Numerous spherical capsules were observed. When comparing the chitosan capsules produced with the two gelling agents, the average diameter and CV were smaller when PTA was used, with 78% of the capsules falling between 20 and 50 nm. On the other hand, when TPP was used, most capsules were around 40 nm, but capsules between 100 and 200 nm also accounted for 22%. The difference in capsule diameter and CV between the two gelling agents can be explained by the gelation rate. Literature has shown that the gelation rate of chitosan with TPP is relatively slow.^25^ This property may cause chitosan droplets to coalesce before the gel stabilizes. As a result, larger particles were also produced, leading to a broader size distribution of the chitosan capsules. In contrast, PTA has a relatively fast gelation rate for chitosan. Therefore, as soon as the chitosan droplets came into contact with PTA, stable gel capsules were produced, resulting in uniform and small-sized capsules. Based on these results, PTA was determined to be the more suitable gelling agent for synthesizing uniform chitosan capsules and was used in subsequent experiments.

**Figure 4:**
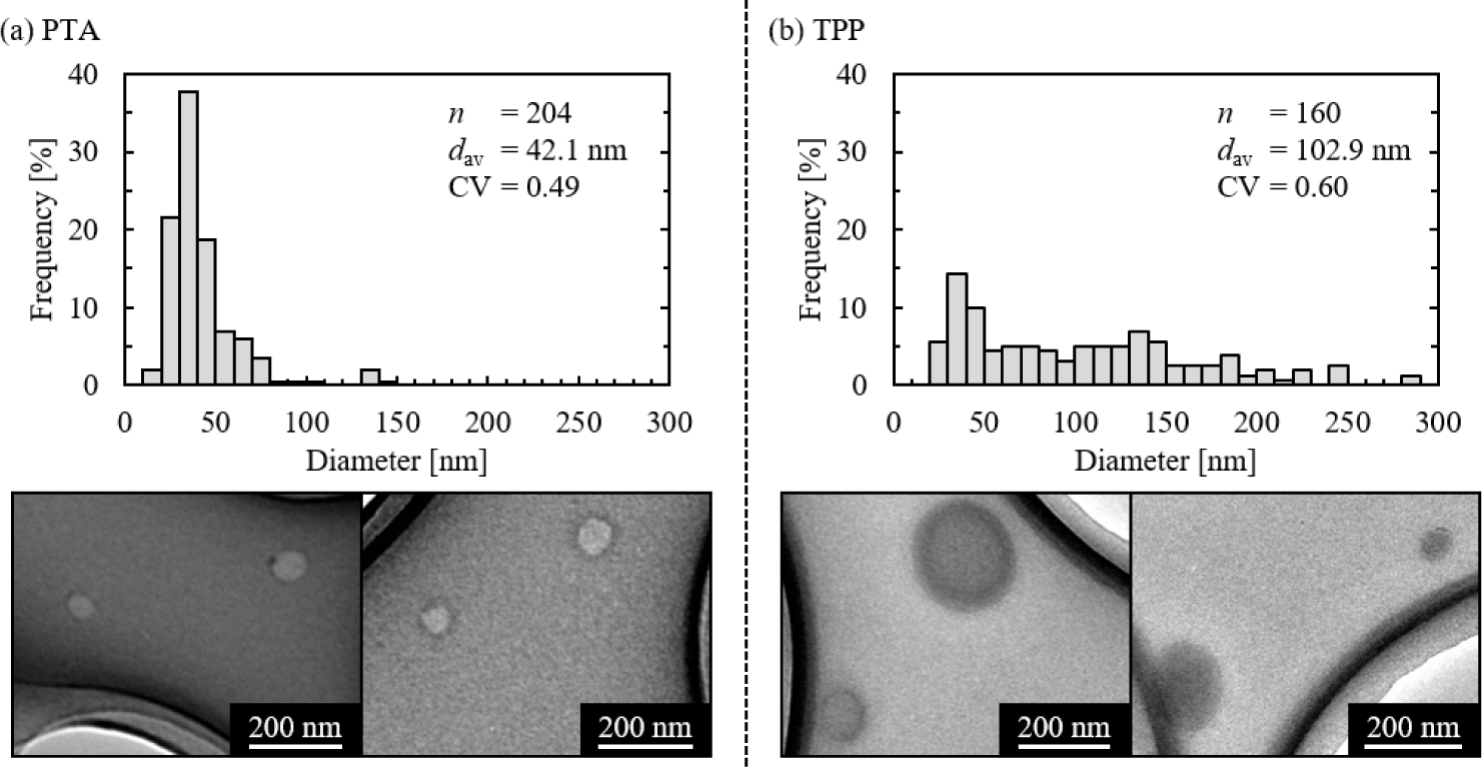
TEM images and particle size distributions of the synthesized chitosan capsules. Gelling agents: (a) PTA and (b) TPP.

### 3.2 Synthesis of Chitosan Gel-Encapsulated AuNPs

Next, the synthesis of chitosan capsules containing AuNPs was conducted. A representative TEM image is shown in Figure 5a. An internal phase containing many AuNPs was observed inside the capsule. Thus, the synthesis of chitosan gel-encapsulated AuNPs was successfully demonstrated. The effect of encapsulation on the optical properties was analyzed by UV–vis–NIR spectrometry, and the absorption spectra are shown in Figure 5b. The absorption peak was observed at about 550 nm, which is generally expressed by AuNPs with a size of 4 nm or larger.^26^ It can be concluded that the optical properties were not disturbed by encapsulation and were sufficiently maintained. Considering that the time gap between the start of reduction and gelation is an important factor, the distance between the collision point and the collection vial was changed from 8.5 to 3 or 15 cm, and experiments were performed. In this case, the time intervals were 0.4, 1.1, and 1.9 s for distances of 3, 8.5, and 15 cm, respectively. The time required to reach terminal velocity was calculated to be 0.0082 s, indicating that the drop velocity was almost constant regardless of position; therefore, the effect of velocity was negligible. The results are shown in Figure 6. Chitosan capsules approximately 350 nm in size were produced, and chitosan gel-encapsulated AuNPs were obtained at distances of 3 and 8.5 cm. At 15 cm, capsules with AuNPs entrapped in the capsule shell were observed, along with numerous aggregates of unencapsulated AuNPs (Figure 6c). The average diameters of the chitosan capsules and internal phases are also summarized in Figure 6. It can be observed that the internal phase became smaller as the time gap increased, leading to a decrease in the encapsulation efficiency of the AuNPs. The diameter of the chitosan capsules was approximately 350 nm under the examined conditions. Based on the results related to the time gap effect, the mixing properties within the falling droplet were speculated as follows, as summarized in Figure 7. When the aqueous solution and polymer solution droplets come into contact, the polymer spreads instantly on the surface of the merged droplet.^27^ In the case of 3 cm (0.4 s), the droplet falls into the gelling agent solution in this state, and the chitosan concentrated on the surface becomes a gel. The large internal phase primarily originated from the reducing agent that contained synthesized AuNPs. In the case of 8.5 cm (1.1 s), the longer time gap allowed chitosan to diffuse into the droplet before gelling, resulting in a smaller internal phase compared to that at 3 cm. In contrast, at 15 cm (1.9 s), diffusion and mixing were completed, and both chitosan and AuNPs were dispersed throughout the droplet before gelling. When this droplet fell into the gelling agent solution and gelation occurred due to the action of PTA from the surface, the AuNPs moved to the droplet surface, as if they were being dragged by the chitosan. As a result, no internal phase was formed, and the synthesized AuNPs were expelled to the capsule shell or even outside the capsule. This behavior was probably caused by the multiple roles of chitosan: as a protective agent for the AuNPs and as a capsule material. It may be possible to create AuNP-dispersed capsules by selecting a protective agent that does not serve as a capsule material.

**Figure 5:**
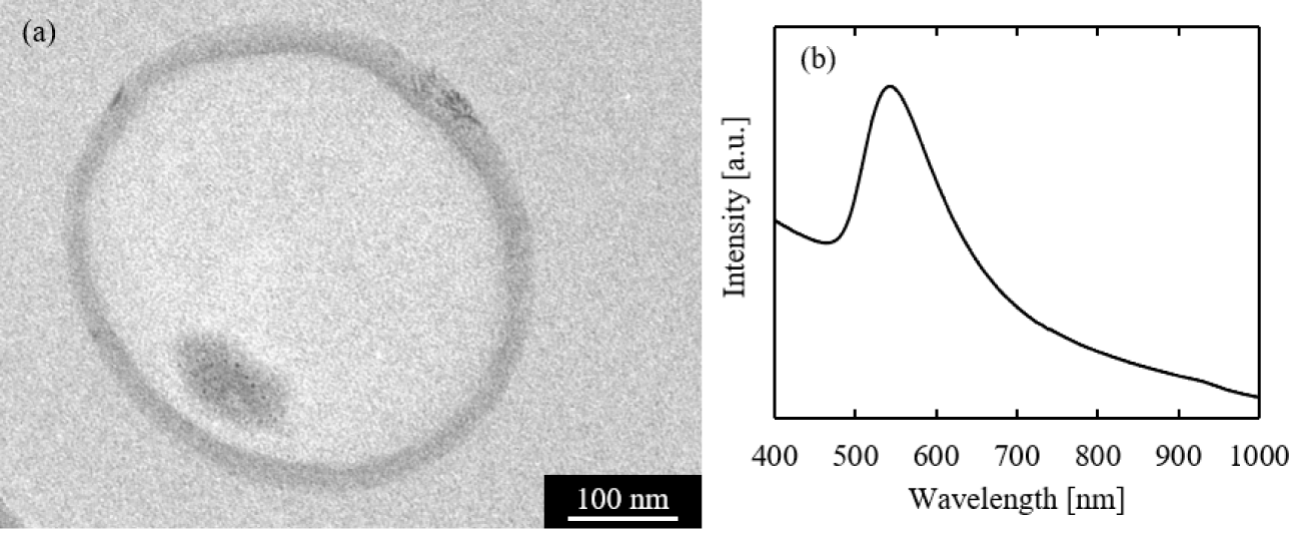
(a) TEM image. (b) UV–vis–NIR absorption spectra of the synthesized chitosan gel-encapsulated AuNPs.

**Figure 6:**
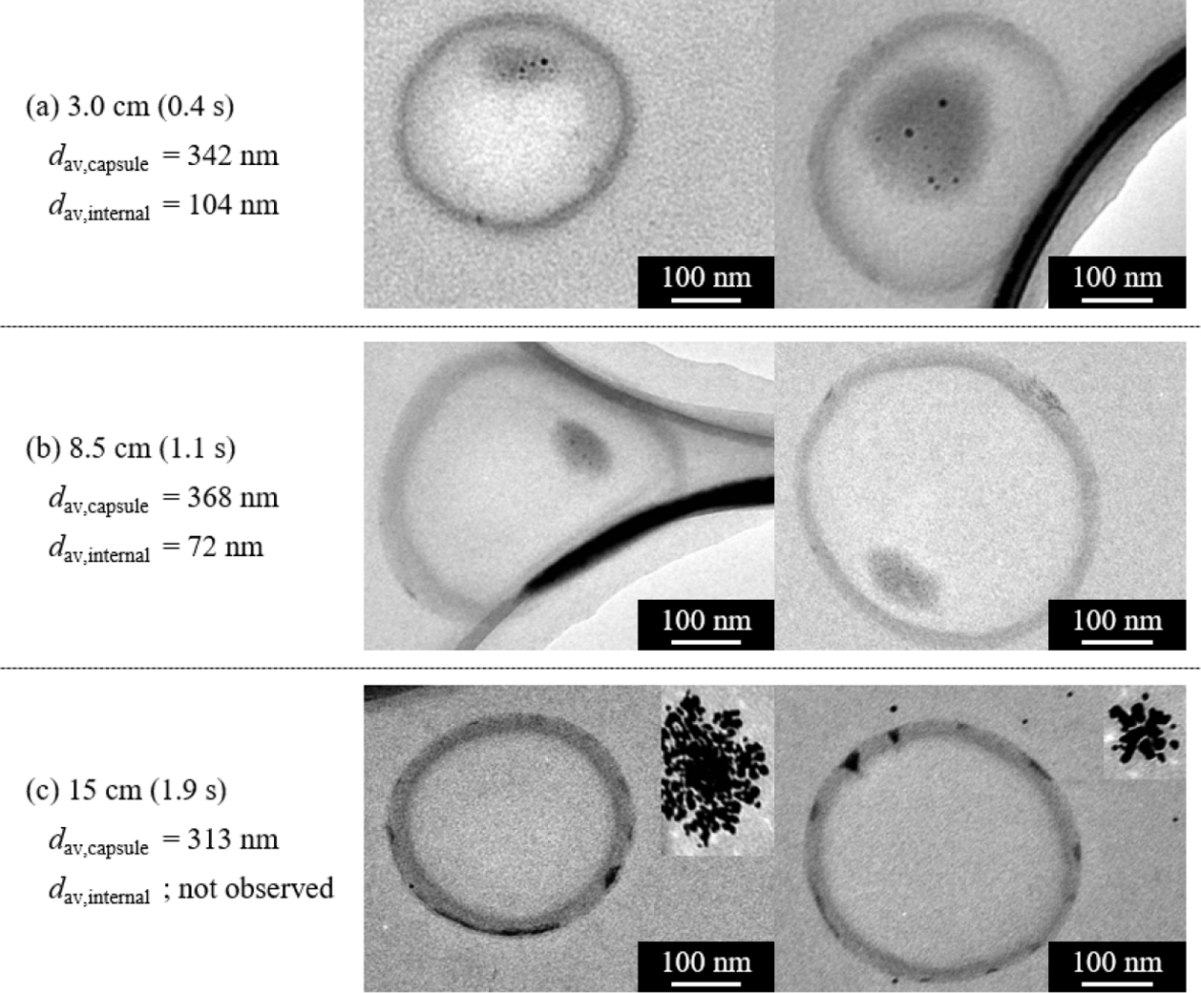
TEM images of the synthesized AuNPs and the chitosan gel samples. Distance between the collision point and the collection vial: (a) 3.0 cm, (b) 8.5 cm, (c) 15 cm.

**Figure 7:**
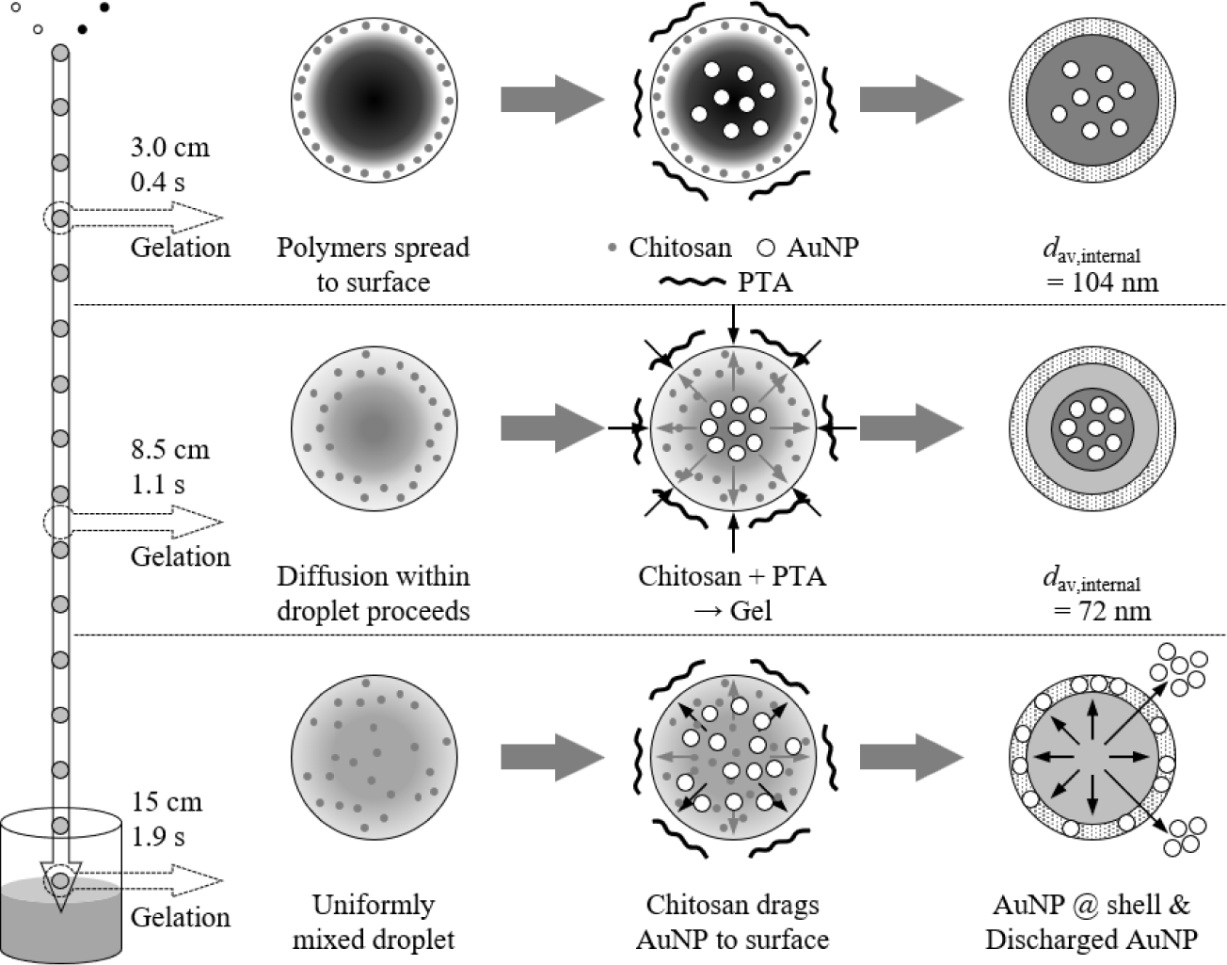
Effect of the reaction time between collision and gelation on the synthesis of chitosan gel-encapsulated AuNPs.

### 3.3 Future Perspectives

One of the major advantages of this method is its high reproducibility. Inkjet printing is a well-established technique, and its precision is widely recognized. In addition, the rapid mixing enabled by the collision of tiny droplets presents another significant benefit. The significance of this study lies in demonstrating the high potential of this precise device through process design. First, uniformly sized AuNPs were synthesized within tiny droplets, which can be regarded as miniature reactors. By stabilizing these droplets as gel capsules, the one-pot synthesis of chitosan gel-encapsulated AuNPs was successfully achieved. This method is applicable to a wide range of nanoparticles, provided that the gel material and nanoparticle precursors do not interfere with each other undesirably. For example, our group has previously reported the production of silver nanoparticles and polymer-complex nanoparticles using the inkjet mixing system.^11,12^ It was demonstrated that particle size and morphology could be controlled by adjusting collision conditions, material concentrations, and solvent properties. The synthesis mechanism for silver nanoparticles, which involves the reduction of silver ions, is essentially the same as that used in this study. This suggests that the type of nanoparticles encapsulated in chitosan gel is not limited to AuNPs. Most previous outstanding research has involved changing the properties of inkjet droplets or the conditions of the collecting side to produce microparticles or capsules.^28–30^ However, by utilizing the high reproducibility of inkjet and employing the collision process, as in this study, further developments in inkjet technology that have not been considered until now are expected. The system can be further enhanced by integrating additional apparatus, such as temperature control units or supplementary nozzles, to enable sequential reactions. However, the system also has drawbacks, with the most significant being low productivity. Due to the extremely small size of the droplets, the processing volume over time is quite limited. This limitation can be addressed through “numbering-up,” or system parallelization. Thanks to its high reproducibility, parallelization can directly improve productivity without compromising product quality. However, implementing parallelization involves high costs and requires considerable space, which is not easily overcome. Therefore, practical applications may be best limited to high-value fields, such as controlled drug delivery systems. For drug delivery applications, verification of stability is essential. Controlling the surface properties of nanoparticles is an effective strategy for enhancing stability.^31^ For example, Temur et al. achieved ultrastability by coating single AuNPs with a dihydrolipoic acid derivative.^32,33^ Sequential reactions could represent an important advancement, potentially enabling the fabrication of encapsulated particles with controlled surface properties. The encapsulation efficiency is also one of the important factors for drug delivery applications because the chemicals to be encapsulated usually require high costs. Encapsulation efficiency could be increased with chitosan concentration when new coccine was encapsulated (see Supporting Information). However, higher chitosan concentration promotes diffusion within the droplet; therefore, the balance between the dragging effect should be considered for the AuNP case. In addition, too much of an increase in chitosan concentration results in a viscosity increase, which spoils the stable droplet discharge from the inkjet nozzles.

## 4 Conclusions

The synthesis of AuNPs and chitosan gel capsules was examined, and a one-pot synthesis of chitosan gel-encapsulated AuNPs was achieved using an inkjet mixing system. Uniform AuNPs with an average diameter of 5.1 nm and a CV of 0.19 were successfully synthesized, demonstrating the advantages of inkjet mixing technology in nanoparticle production, such as rapid mixing and highly reproducible tiny droplet ejection. Chitosan capsules were synthesized by dripping a chitosan solution into a gelling agent. The size and dispersion of the capsules produced changed depending on the difference in the gelation rate of the gelling agent. Chitosan gel-encapsulated AuNPs were synthesized using a one-pot method with inkjet technology. The effect of the time between AuNP nucleation and chitosan gelation was examined, and it was demonstrated that a shorter time gap was desirable for the production of chitosan gel-encapsulated AuNPs.

## Supplementary Material



## Data Availability

No data were used for the research described in the article.

## Notes

Effect of chitosan concentration on encapsulation efficiency (PDF)

## Abbreviations

AuNP: gold nanoparticle
PTA: phosphotungstic acid
TPP: sodium tripolyphosphate
TEM: transmission electron microscope
CV: coefficient of variation
