# Supplementary material for: A One-Pot Fabrication of Chitosan Gel-Encapsulated Gold Nanoparticles Using Inkjet Mixing Technology
Source: ACS Omega. 2025 Sep 4;10(36):41214–20. doi: 10.1021/acsomega.5c03957 (PMC12444684; doi:10.1021/acsomega.5c03957)
Supplement: Supplementary file 1 [file ao5c03957_si_001.pdf]

Supporting Information:

One-pot fabrication of chitosan gel encapsulated gold nanoparticles using inkjet mixing technology

*Yosuke Muranaka, Yukako Nishimuro, Taisuke Maki**

Department of Chemical Engineering, Kyoto University, Kyoto, 615-8510, Japan

*Corresponding author: tmaki@cheme.kyoto-u.ac.jp

This Supporting Information provides the investigation results of encapsulation efficiency, examined using new coccine as a model material to be encapsulated. new coccine is known as a red food coloring agent. The reason why new coccine was selected as a model material was, it was easy to analyze the encapsulation efficiency by just analyzing the absorbance of the supernatant.

MATERIALS AND METHODS

A 1 mM tetrachloroauric(III) acid was replaced with 2.4 g/L new coccine, which was purchased from Fujifilm Wako Chemical (Japan). Chitosan concentration was prepared as 15 mM or 30 mM. All the other procedures were the same as the chitosan gel encapsulated AuNPs production case. After collecting the determined amount of droplets, the absorbance by supernatant at the wavelength of 503 nm was analyzed using UV-vis-NIR spectrophotometer (V-730, JASCO, Japan). Encapsulation efficiency η was calculated by **Equation S1**.

$$\eta = 1 - C/C_0 \qquad \textbf{– Equation S1}$$

C is the concentration of new coccine in supernatant, and C_0 is the concentration of new coccine without gel materials.

RESULTS

Encapsulation efficiency η was 0.66 and 0.93 for 15 mM chitosan and 30 mM chitosan, respectively. The encapsulation efficiency increased with the chitosan concentration. **Figure S1** shows the photo of the product when 15 mM chitosan was used.

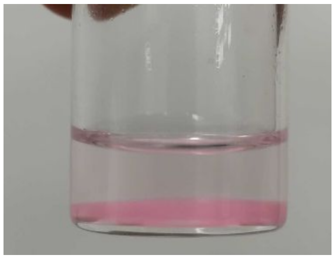

Figure S1. Chitosan gel encapsulated new coccine. Chitosan concentration was 15 mM.
